# Extrakorporale Verfahren bei Vergiftungen

**DOI:** 10.1007/s00063-024-01156-6

**Published:** 2024-07-10

**Authors:** Gerald Hackl, Nikolaus Schreiber

**Affiliations:** 1 Allgemeine Intensivstation, Universitätsklinik für Innere Medizin, Medizinische Universität Graz, Auenbruggerplatz 15, 8036 Graz, Österreich; 2 Klinische Abteilung für Nephrologie, Universitätsklinik für Innere Medizin, Medizinische Universität Graz, Auenbruggerplatz 27, 8036 Graz, Österreich

**Keywords:** Renale Dialyse, Plasmapherese, Hämofiltration, Hämoperfusion, Toxikokinetik, Renal dialysis, Plasmapheresis, Hemofiltration, Hemoperfusion, Toxicokinetics

## Abstract

In seltenen Fällen benötigen PatientInnen mit lebensbedrohlichen Intoxikationen ein extrakorporales Verfahren zur erweiterten Giftelimination. Die Extracorporeal Treatments in Poisoning (EXTRIP) Workgroup bietet konsens- und evidenzbasierte Empfehlungen mit laufender Aktualisierung bezüglich des Einsatzes von extrakorporalen Verfahren im Management von kritisch kranken, vergifteten PatientInnen. Die extrakorporale Clearance ist am höchsten bei niedermolekularen Substanzen mit niedrigem Verteilungsvolumen, niedriger Plasmaproteinbindung und hoher Wasserlöslichkeit. Um den Effekt der extrakorporalen Clearance zu maximieren, sollten Blut- und Dialysatfluss so hoch wie möglich sein und die Membran mit der größten Oberfläche verwendet werden. Meistens kommt aufgrund der höchsten Effektivität die intermittierende Hämodialyse zur Anwendung, wobei hämodynamisch kompromittierte PatientInnen von einem kontinuierlichen Verfahren profitieren können.

## Abstract (en)

In rare cases, intoxicated patients may require an extracorporeal procedure for enhanced toxin elimination. The Extracorporeal Treatments in Poisoning (EXTRIP) workgroup provides consensus- and evidence-based recommendations regarding the use of extracorporeal procedures in the management of critically ill, poisoned patients, with ongoing updates. Extracorporeal clearance is highest for low molecular weight substances with low volume of distribution, low plasma protein binding, and high water-solubility. To maximize the effect of extracorporeal clearance, blood and dialysate flow rates should be as high as possible, and the membrane with the largest surface area should be utilized. Intermittent hemodialysis is the most commonly employed extracorporeal procedure due to its highest effectiveness, while hemodynamically compromised patients can benefit from a continuous procedure.

## Lernziele

Nach der Lektüre dieses Beitrags …

- beurteilen Sie das Risiko im Rahmen von Intoxikationen effizient und zielführend;
- wissen Sie, wo evidenzbasierte Empfehlungen zum Einsatz von extrakorporalen Verfahren im Rahmen von Intoxikationen zu finden sind;
- kennen Sie ausschlaggebende Faktoren, die die extrakorporale Elimination eines Gifts beeinflussen;
- können Sie ausgehend von den physikochemischen Eigenschaften einer Substanz die Dialysierbarkeit abschätzen und wissen, welche Verfahren zu Verfügung stehen.

## Hintergrund

Der Einsatz von extrakorporalen Verfahren im Rahmen von Vergiftungen ist von Unsicherheiten, Kontroversen und Debatten geprägt, kann mitunter aber lebensrettend sein. Im Verlauf des letzten Jahrzehnts wurden vermehrt **evidenzbasierte Konsensusempfehlungen**

evidenzbasierte Konsensusempfehlungen

zum sinnvollen Einsatz von extrakorporalen Eliminationsverfahren publiziert. Dieser CME-Artikel soll eine **praxisbezogene Übersicht**

praxisbezogene Übersicht

der theoretischen Rationale hinter extrakorporalen Verfahren im Rahmen der Toxikologie geben, um insbesondere für den Fall, dass keine Empfehlungen zur Verfügung stehen, Grundlagen für toxikokinetische und individuelle therapeutische Überlegungen zu schaffen.

Vergiftungen stellen weltweit eine bedeutsame Ursache für Morbidität und Mortalität dar. Die Evidenz bezüglich spezifischer Therapieempfehlungen bei Intoxikationen stützt sich jedoch zumeist auf Fallberichte und retrospektive Beobachtungsstudien, da es an prospektiven randomisierten kontrollierten Studien im Rahmen der Toxikologie mangelt.

Die zentralste und wichtigste Säule in der Behandlung von Intoxikationen stellt die **symptomatische Behandlung**

symptomatische Behandlung

mit Sicherung der Vitalfunktionen dar. Bei den **Gifteliminationsverfahren**

**Abb. 1:**
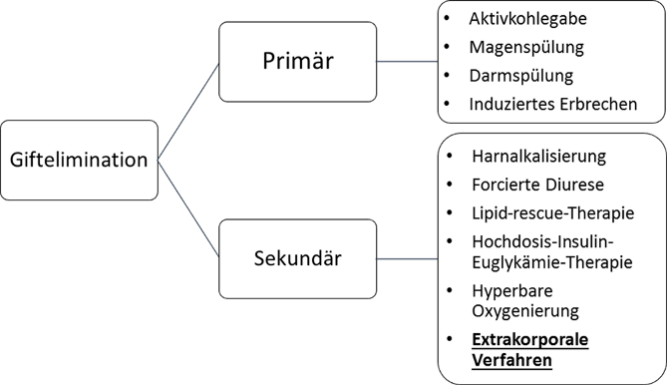
Maßnahmen zur primären und sekundären Giftelimination

Gifteliminationsverfahren

wird grundsätzlich eine primäre von einer sekundären Giftelimination unterschieden. Die primäre Giftelimination hat die Vermeidung der Giftaufnahme in den Körper zum Ziel. Bei der sekundären Giftelimination wird ein bereits im Körper befindliches Gift rascher wieder eliminiert. Die extrakorporale Toxinelimination ist daher den sekundären Gifteliminationsmaßnahmen zuzuordnen (Abb. 1).

Prinzipiell kommen extrakorporale Verfahren einerseits zur sekundären Giftelemination, andererseits aber auch zur Therapie von konsekutiv aufgetretenen Komplikationen von gewissen Intoxikationen, wie z. B. des akuten Nierenversagens, zur Anwendung.

Die Durchführung einer **extrakorporalen Toxinelimination**

extrakorporalen Toxinelimination

erscheint nur relativ selten sinnvoll indiziert und wird von Vergiftungsinformationszentralen in nur < 0,5 % der Fälle empfohlen. Eine rezente Arbeit aus Deutschland zeigte z. B., dass extrakorporale Verfahren bei Intoxikationen nur in 1,9 % der Fälle notwendig wurden, wobei in 95 % der Fälle eine intermittierende Hämodialyse (iHD) das Verfahren der Wahl darstellt [1, 2].

## Grundsätzliche Überlegungen in der Evaluierung der therapeutischen Strategie

### Risikobeurteilung

Intoxikationen, seien sie akzidentiell oder in suizidaler Absicht, entstehen zumeist durch Exposition gegenüber Medikamenten, Drogen, Chemikalien, tierischen oder pflanzlichen Giften. Ferner können Intoxikationen akute, chronische oder **akut-auf-chronische Verläufe**

akut-auf-chronische Verläufe

annehmen und sich in Abhängigkeit von Prädisposition und Vorerkrankungen sehr divergent manifestieren [3]. Dies hat zur Folge, dass jedes klinische Szenario eines individuellen Therapieansatzes bedarf.

Gewisse Intoxikationen (insbesondere durch Monosubstanzen) können sich in Form von typischen Symptomkonstellationen (**Toxidrome**

Toxidrome)

manifestieren. Sie können hilfreich sein, um die Ursache der vermuteten Intoxikation zu identifizieren. Untersuchungen, wie ein Routine-EKG, eine Blutgasanalyse und ein Standardlabor (inkl. Nierenfunktionsparameter sowie Bestimmung der Anionenlücke), sind zur weiteren Risikobeurteilung essenziell. Auch **Spiegelbestimmungen**

Spiegelbestimmungen

gewisser Substanzen, sofern möglich, können eine wichtige Rolle für die weitere Risikoeinschätzung spielen.

Die **spezifische Toxizität**

spezifische Toxizität

einer Substanz resultiert aus einem komplexen Zusammenspiel ihrer intrinsischen Eigenschaften, aber auch aus der Dosis, der Formulierung, der Applikationsart und der individuellen Prädisposition der jeweiligen Person [4].

Nach oder bereits während der Stabilisierung der Vitalfunktionen von lebensbedrohlich vergifteten PatientInnen sollte begonnen werden, den weiteren klinischen Verlauf zu antizipieren und das Risiko für einen schwerwiegenden Verlauf zu beurteilen. Essenzielle Bestandteile der Risikoeinschätzung sind die Identifikation der **ursächlichen Substanz**

ursächlichen Substanz

(was?), die Menge (wieviel?), patientInnenbezogene Faktoren und individuelle Prädisposition (wer?) sowie der zeitliche Kontext (wann?). Ziel dieser initialen Risikobeurteilung ist es, die Wahrscheinlichkeit von signifikanten Folgen nach einer spezifischen Exposition eines Giftes zu beurteilen. Ein integraler Bestandteil in diesem Prozess ist die anfängliche Evaluierung alternativer therapeutischer Möglichkeiten, um Toxizität vorzubeugen, zu reduzieren oder zu reversieren [3]. Im Rahmen dieses Prozesses muss das Vorhandensein **spezifischer Antidote**

spezifischer Antidote,

aber auch absorptionspräventiver Maßnahmen wie Aktivkohle oder aber der Möglichkeit einer forcierten Elimination evaluiert werden. Wenn das Risiko für eine **lebensbedrohliche Vergiftung**

lebensbedrohliche Vergiftung

oder eine damit im Zusammenhang stehende Komplikation anzunehmen ist und gleichzeitig keine effektivere Behandlungsalternative zu Verfügung steht, sollte ein extrakorporales Verfahren in Betracht gezogen werden.

#### Merke

Am Beginn des Evaluierungsprozesses zum Einsatz eines extrakorporalen Verfahrens steht ein strukturiertes Risikoassessment.

### Extracorporeal Treatments in Poisoning Workgroup

Die Extracorporeal Treatments in Poisoning (EXTRIP) Workgroup stellt eine multinationale und **interdisziplinäre Arbeitsgruppe**

interdisziplinäre Arbeitsgruppe

aus anerkannten Experten aus den Bereichen der Nephrologie, Pharmakologie, Toxikologie und Methodik dar, um systematisch und in regelmäßigen Abständen die verfügbare Literatur zum sinnvollen Einsatz von extrakorporalen Verfahren bei Intoxikationen zu sichten, relevante Informationen zu extrahieren, zusammenzufassen sowie evidenzbasierte Empfehlungen zu veröffentlichen [5].

Die Handlungsempfehlungen der EXTRIP Workgroup sind klar strukturiert und entsprechend dem Empfehlungsgrad bewertet, weshalb sie in den meisten Fällen eine praxisnahe Hilfestellung bieten. Aktuelle **Leitlinien**

Leitlinien

und Empfehlungen sind auf ihrer Homepage abrufbar [6].

## Ausschlaggebende Determinanten bei fehlender evidenzbasierter Empfehlung

Die folgenden Ausführungen sollen dabei unterstützen, den potenziellen Nutzen eines extrakorporalen Verfahrens in der Therapie einer schweren Intoxikation zu evaluieren, wenn keine evidenzbasierte Entscheidungshilfe oder Empfehlung zur Verfügung steht.

Ob ein Gift einer Elimination durch extrakorporale Verfahren zugänglich ist, hängt maßgeblich von den physikochemischen und **toxikokinetischen Eigenschaften**

toxikokinetischen Eigenschaften

der zu eliminierenden Substanz ab. Die ausschlaggebenden Determinanten sind hierbei das Molekulargewicht, das Verteilungsvolumen bzw. die Wasser‑/Fettlöslichkeit, die Proteinbindung und die endogene Clearance.

### Molekulargewicht

Ein Gift kann nur durch ein extrakorporales Verfahren entfernt werden, wenn es größentechnisch durch die Poren einer extrakorporalen Membran passt. Daher gilt, je kleiner eine Substanz bzw. deren Molekulargewicht, umso wahrscheinlicher ist diese auch dialysierbar. Die meisten Gifte im klinischen Alltag haben ein Molekulargewicht < 2 kDa. Typischerweise besitzen Dialysemembranen für die iHD molekulare Cut-off-Werte von 10(–15) kDa. Mittels spezieller **High-cut-off-Membranen**

High-cut-off-Membranen

sind sogar Substanzen mit Molekulargewichten von 50(–60) kDa entfernbar. Daher stellt das Molekulargewicht eines Toxins häufig keine Kontraindikation zur Elimination dar [3]. Gifte, die höhere Molekulargewichte besitzen (z. B. monoklonale Antikörper) können nur durch **Plasmaphereseverfahren**

Plasmaphereseverfahren

eliminiert werden, da hierbei ohnehin das Gesamtplasmavolumen ausgetauscht wird und das Molekulargewicht daher keine Rolle spielt. Durch die Addition der Atommassen eines Moleküls kann die **selbstständige Berechnung**

selbstständige Berechnung

des Molekulargewichts relativ einfach erfolgen. Beispiel: Es liegt eine Methanol(CH_3_OH)-Intoxikation vor. Anhand der Verwendung eines Periodensystems der Elemente kann die Atommasse für jedes einzelne Element des Moleküls abgelesen werden. Durch Addition aller Atommassen ergibt sich für Methanol ein Molekulargewicht von 1 × 12,011 (C-Atom) + 4 × 1,0079 (4 H-Atome) + 1 × 15,999 (1 O-Atom) = 32,0416 Da.

#### Merke

Das Molekulargewicht ergibt sich aus der Addition aller Atommassen eines Moleküls: mM = n_1_ × mA_1_ + n_2_ × mA_2_ + n_3_ × mA_3_ + ….

### Verteilungsvolumen

Um eine Substanz mittels extrakorporaler Verfahren aus dem Körper eliminieren zu können, sollte sich der Großteil der Substanz im **intravasalen Raum**

intravasalen Raum

befinden, da nur dieser einem extrakorporalen Verfahren zugänglich ist. Das Verteilungsvolumen stellt eine theoretische Größe dar und ist ein Maß für die Wirkstoffverteilung im extravasalen Raum bzw. für das Verhältnis der Giftmenge im Körper zur Konzentration im Plasma oder Blut [7]. Es wird primär von der **Lipophilie**

Lipophilie

der zu entfernenden Substanz bestimmt: je höher die Lipophilie, umso größer ist das Verteilungsvolumen; je höher die **Hydrophilie**

Hydrophilie,

umso kleiner ist das Verteilungsvolumen. Daher können grundsätzlich lipophile Substanzen schlecht, und hydrophile Substanzen gut mittels extrakorporaler Verfahren aus dem Körper entfernt werden [5]. Als Grenze wird in Abhängigkeit des gewählten Verfahrens ein Verteilungsvolumen von ≤ 1 l/kgKG (maximal 2 l/kgKG) betrachtet [3]. In gewissen Situationen kann bei Substanzen mit sehr hohem Verteilungsvolumen (z. B. Methotrexat) eine schnellstmögliche extrakorporale Elimination sinnvoll sein, noch bevor **Distributionsphänomene**

Distributionsphänomene,

z. B. in das Fettgewebe, stattgefunden haben [8]. Um in der Praxis das Verteilungsvolumen näherungsweise zu berechnen, ist die Kenntnis der eingenommenen Dosis sowie eine Spiegelbestimmung Voraussetzung. Beispiel: Wenn ein Medikament einen **Plasmaspiegel**

Plasmaspiegel

von 20 mg/l aufweist und insgesamt 1000 mg eingenommen wurden, beträgt das Verteilungsvolumen 50 l. Bei Korrektur auf das Körpergewicht besteht bei 70 kgKG ein Verteilungsvolumen von 50 l /70 kgKG = 0,71 l/kgKG, weshalb die Substanz zum gegebenen Zeitpunkt im Hinblick auf das Verteilungsvolumen als dialysierbar einzustufen wäre.

#### Merke

Verteilungsvolumen [l/kgKG] = eingenommene Dosis [mg]/Plasmakonzentration [mg/l]/Körpergewicht [kg].

### Proteinbindung

Ein weiterer entscheidender Faktor ist die Plasmaproteinbindung. Dabei handelt es sich um das Ausmaß der Bindung einer Substanz an Eiweiße im Plasma (im Wesentlichen an **Albumin**

Albumin).

Mit Ausnahme der Plasmapherese ist nur die frei im Plasma vorhandene Fraktion einer Substanz der extrakorporalen Entfernung zuführbar, da der **Toxin-Protein-Komplex**

Toxin-Protein-Komplex

(z. B. bei Bindung an Albumin) zu groß ist (> 65 kDa), um die Poren einer Dialysemembran passieren zu können.

Als Faustregel gilt, dass Gifte, die mehr als 80 % Plasmaproteinbindung aufweisen, schlecht dialysierbar sind [3]. Da die Fähigkeit der Eiweißbindung einem Sättigungsverhalten unterliegt, kann diese im toxischen Spiegelbereich deutlich abnehmen. Dadurch fällt vermehrt freies Toxin an, das schließlich mit extrakorporalen Verfahren entfernt werden kann. Die **Pharmakokinetik**

Pharmakokinetik

entspricht also nicht unbedingt der Toxikokinetik. Dies trifft etwa auf Salizylate, Valproat oder Carbamazepin zu [9]. Praxistipp: Das Ausmaß der Plasmaeiweißbindung von Medikamenten wird etwa in der Fachinformation unter den pharmakologischen bzw. pharmakokinetischen Eigenschaften angegeben. Ansonsten sollte diesbezüglich auch im Internet recherchiert sowie Rücksprache mit Vergiftungsinformationszentralen gehalten werden.

### Endogene Clearance

Eine weitere wichtige Überlegung stellt die Berücksichtigung der körpereignen (endogenen) Clearance einer Substanz dar, die sich im Wesentlichen aus der Summe von renalen und nichtrenalen (vorwiegend hepatischen) Clearance ergibt, und prinzipiell jenes Plasmavolumen beschreibt, aus dem eine Substanz in einer bestimmten Zeit entfernt wird [10].

Um den Einsatz eines extrakorporalen Verfahrens zur Giftelimination zu rechtfertigen, muss dieses erheblich zur **Gesamtclearance**

Gesamtclearance

einer Substanz beitragen. Realistischerweise kann die extrakorporale Clearance etwa 200(–300) ml/min erreichen [3]. Beträgt die endogene Clearance z. B. durch Metabolismus in der Leber 1000 ml/min, kann die extrakorporale Clearance nicht signifikant zur Gesamtclearance beitragen und ein extrakorporales Verfahren ist nicht gerechtfertigt. Bei Giften, die hauptsächlich renal eliminiert werden, ist die endogene Clearance im Rahmen einer akuten (oder chronischen) **Nierenschädigung**

Nierenschädigung

verringert [8]. Dies vergrößert wiederum den relativen Anteil der extrakorporalen Clearance an der Gesamtclearance. Die endogene (hauptsächlich renal bedingte) Clearance von Metformin beträgt z. B. unter der Annahme einer normalen Nierenfunktion etwa 500–600 ml/min, während diese im Fall einer akuten Nierenschädigung auf < 5 ml/min absinken kann [11]. Dadurch wird zumindest in Abhängigkeit der Clearance der Vorteil einer extrakorporalen Entfernung deutlich (sofern die Kriterien einer metforminassoziierten Laktatazidose, wie seitens der EXTRIP Workgroup empfohlen, erfüllt sind). Für die Praxis kann ein **pragmatischer Ansatz**

pragmatischer Ansatz

gewählt werden. Bei Vorliegen einer unauffälligen Nieren- und/oder Leberfunktion sollte ein extrakorporales Verfahren eher nicht in Betracht gezogen werden.

#### Merke

Unter Clearance versteht man jenes Plasmavolumen, aus dem eine Substanz in einem bestimmten Zeitraum entfernt wird.

#### Merke

Molekulargewicht, Verteilungsvolumen, die Wasser- bzw. Fettlöslichkeit, die Proteinbindung und die endogene Clearance sind die entscheidenden Determinanten, von denen die Effektivität eines extrakorporalen Therapieverfahrens abhängt.

Die EXTRIP Workgroup schlägt zur Orientierung und zur Bestimmung der Effektivität von extrakorporalen Verfahren im Rahmen der sekundären Giftelimination folgende **Kriterien**

Kriterien

vor: Werden im Rahmen einer 6‑stündigen Behandlungsdauer eines extrakorporalen Verfahrens mehr als 30 % der ursprünglich ingestierten Dosis einer Substanz eliminiert, wird die Substanz, als dialysierbar bezeichnet. Liegt der Bereich zwischen 10 und 30 %, ist die Substanz moderat dialysierbar, zwischen 3 und 10 % eingeschränkt dialysierbar und unter 3 % nichtdialysierbar [5].

In Tab. 1 sind zur schnellen Übersicht Substanzen, die in häufigen Fällen zu Intoxikationen führen, bezüglich ihrer Dialysierbarkeit gegenübergestellt.

**Tab. 1:** Effektiv bzw. nichteffektiv dialysierbare Substanzen

Effektiv dialysierbar	Nichteffektiv dialysierbar
Lithium	Digoxin
Methanol	Trizyklische Antidepressiva
Ethylenglykol	Phenytoin
Salizylate	β‑Blocker
Barbiturate	Benzodiazepine
Metformin	Makrolide
Aminoglykoside, Metronidazol	Fluorchinolone
Carbapeneme, Penizilline, Cephalosporine	Warfarin

## Extrakorporale Verfahren

Prinzipiell kann man die Clearanceprinzipien mittels extrakorporaler Verfahren in 4 verschiedene Kategorien einteilen, die auf unterschiedlichen **Stofftransportmechanismen**

Stofftransportmechanismen

basieren. Man unterscheidet zwischen diffusiven, konvektiven, adsorptiven und zentrifugalen Clearanceprinzipien.

Ausgehend von diesen Konzepten wird zwischen Hämodialyse (HD), Hämofiltration (HF), Hämoperfusion (HP) und Plasmapherese (PPH) differenziert.

Bei der HD kommt die **diffusive Clearance**

diffusive Clearance

zum Tragen, während bei der HF das Prinzip der Konvektion ursächlich für die Elimination ist. Im Rahmen der Hämodiafiltration (HDF) liegt gegebenenfalls ein Vorteil in der Erhöhung der Eliminationsrate durch eine Kombination dieser beiden Prinzipien. Bei der HP wird über die **Adsorption**

Adsorption

eliminiert und die PPH nutzt das Prinzip der Zentrifugation. Die Bedeutung der Membraneffizienz sowie des Flusses wird in der Infobox 1 näher erläutert.

### Infobox Was bedeutet Effizienz („efficiency“) bzw. Fluss („flux“) in Bezug auf die Membran?

Die Fähigkeit einer Dialysemembran, gelöste Stoffe mit niedrigem Molekulargewicht, wie z. B. Harnstoff, zu entfernen, ist von dem Produkt aus Membranoberfläche und der Membranpermeabilität abhängig [12]. Eine High-efficiency-Membran ist grundlegend ein großer Filter mit großer Membranoberfläche. High-efficiency-Membranen können größere oder kleine Poren haben und deshalb hohe oder niedrige Clearanceraten für höhermolekulare Substanzen aufweisen. Die sog. High-flux-Membranen haben große Poren, die es ermöglichen, Moleküle mit höherem Molekulargewicht zu filtern. High-flux-Membranen haben typischerweise auch eine hohe Permeabilität für Wasser. Die Permeabilität für Wasser wird mittels des Ultrafiltrationskoeffizienten angegeben. Dieser beschreibt das Volumen an Plasmawasser in Milliliter, das pro Stunde und Transmembrandruck abfiltriert wird [12].

### Diskontinuierliche Hämodialyse

Während der iHD diffundiert das Gift entlang eines **Konzentrationsgradienten**

Konzentrationsgradienten

aus dem Plasma durch eine semipermeable Membran in ein Dialysat, dessen Strömungsrichtung der des Bluts entgegengesetzt ist [4]. Die iHD erlaubt dabei hohe Dialysat- und Blutflüsse und bietet daher einige Vorteile: Gifte können schnell eliminiert, der Säure-Basen-Haushalt sowie Elektrolytstörungen schnell korrigiert und je nach Wasserhaushalt auch Flüssigkeit entzogen werden. Neben der Verfügbarkeit, schnellen Implementierung sowie der Kosteneffizienz können mit der iHD die **höchsten Clearanceraten**

höchsten Clearanceraten

erreicht werden [4]. Aus diesen Gründen stellt die iHD auch das bevorzugte extrakorporale Eliminationsverfahren für den Großteil der Vergiftungen dar [8]. Nachteilig kann das Auftreten eines **Dysäquilibriumsyndroms**

Dysäquilibriumsyndroms

sein, das bei dialysenaiven Personen aber eher unwahrscheinlich ist, sowie die hämodynamische Belastung aufgrund der hohen Blutflüsse. Beachtet werden sollte unter anderem die Konstitution des Dialysats, sodass z. B. der Zusatz von Phosphat notwendig werden kann.

Das in Deutschland sehr häufig verwendete **Tanknierensystem**

Tanknierensystem,

das sich durch Mobilität aufgrund der Unabhängigkeit von einem Ringleitungssystem und einer individuellen Dialysatzubereitung auszeichnet, wurde bereits nachweislich effektiv im Rahmen von akuten Intoxikationen angewendet [13, 14, 15].

### Hämoperfusion

Die Hämoperfusion nutzt das Prinzip der Adsorption und wurde in den 1970er-Jahren in der Behandlung von Intoxikationen zeitweise sogar präferiert [8]. Der theoretische Vorteil liegt darin, dass die Adsorption weniger von Molekulargewicht und Proteinbindung beeinflusst wird als die Diffusion. Das am häufigsten eingesetzte **Adsorbens**

Adsorbens

ist hierbei Aktivkohle. Nachteile der Hämoperfusion bestehen im Bedarf einer potenteren **Antikoagulation**

Antikoagulation

im Vergleich zu herkömmlichen Dialyseverfahren und darin, dass nur niedrige Blutflüsse verwendet werden können, um das Risiko einer Hämolyse so gering wie möglich zu halten [3]. Ferner werden nichtselektiv Thrombozyten, Kalzium und Glukose adsorbiert, wohingegen sowohl Alkohole als auch Metalle nicht adsorbiert werden. Außerdem muss der **Kohlefilter**

Kohlefilter

aufgrund der Filtersättigung etwa alle 2 h getauscht werden, wodurch das Verfahren relativ teuer ist. Nicht nur deswegen, sondern vor allem auch weil moderne **High-flux-Hämodialysefilter**

High-flux-Hämodialysefilter

eine wirksamere, ubiquitär verfügbare und kosteneffizientere Lösung darstellen, hat die Hämoperfusion zunehmend an Stellenwert verloren. In den rezentesten Empfehlungen der EXTRIP Workgroup findet sich keine Empfehlung zur Kohleadsorption [3].

Auch die US-amerikanischen Zahlen aus dem Jahr 2022 belegen nochmals den schwindenden Gebrauch der Hämoperfusion [2].

Eine vorwiegend in Europa durchgeführte Form der Hämoperfusion ist die Zytokinadsorption mittels eines Adsorbers aus kleinen geladenen, porösen **Polymer-Sorbent-Kügelchen**

Polymer-Sorbent-Kügelchen

mit einer Oberfläche von 45.000 m^2^ [16]. Diese können relativ einfach in Dialysekreisläufe oder auch im Rahmen der extrakorporalen Membranoxygenierung (ECMO) integriert werden und adsorbieren vorwiegend hydrophobe Substanzen mit Molekulargewichten bis 60 kDa [17]. In der Europäischen Union ist das System zwar zur Entfernung von Zytokinen, Myoglobin, Bilirubin, Ticagrelor und auch Rivaroxaban zugelassen, es fehlt allerdings an Evidenz bezüglich seines Einsatzes [18].

### Hämofiltration

Ein weiteres Verfahren stellt die auf Konvektion beruhende HF dar. Hierbei kommt es via eines **Druckgradienten**

Druckgradienten

und Ersatz mittels einer physiologischen Lösung zur Toxinelimination [1]. Grundsätzlich können mittels HF Substanzen mit einer Molekülmasse bis zu 25(–50) kDa entfernt werden. Da der Großteil der bislang bekannten Toxine wie bereits erwähnt eine Molekülmasse von < 2 kDa besitzt, bietet die HF keine wesentlichen Vorteile im Vergleich zur iHD. Aus diesem Grund wird auch dieses Verfahren kaum zur Toxinelimination eingesetzt. Mögliche Indikation wäre die Kombination von iHD und HF im Sinne einer HDF zur Behandlung einer häufig im Rahmen von Intoxikationen begleitenden **Rhabdomyolyse**

Rhabdomyolyse

[19].

Die HF kann auch in Form eines kontinuierlichen Verfahrens zur Anwendung kommen (CVVH) und bietet ebenfalls eine Option zur Toxinelimination.

### Kontinuierliche Verfahren

Kontinuierliche Nierenersatzverfahren, wie die kontinuierliche venovenöse Hämodialyse (CVVHD) oder die kontinuierliche venovenöse Hämodiafiltration (CVVHDF) finden ihren Einsatz vor allem in der Behandlung der **Hypervolämie**

Hypervolämie

oder von Stoffwechsel- bzw. Elektrolytstörungen im Rahmen einer akuten Nierenschädigung im intensivmedizinischen Bereich [4]. Ein Vorteil bei hämodynamisch kompromittierten PatientInnen ist, dass die **Nettoultrafiltrationsraten**

Nettoultrafiltrationsraten

aufgrund der kontinuierlichen Anwendung reduziert werden können [3]. Andererseits ist jedoch aufgrund der niedrigeren Blut- und Dialysatflussraten auch die Toxinclearance um 50–80 % geringer als bei der iHD [8]. Kontinuierliche Verfahren sind allerdings theoretisch in der Lage, redistributionsbedingte **Plasmakonzentrationsanstiege**

Plasmakonzentrationsanstiege

bzw. Rebounds von Toxinen zu minimieren. Diese können unter Umständen nach der schnellen Elimination durch diskontinuierliche Verfahren auftreten, wie es etwa bei Lithiumintoxikationen der Fall sein kann [3].

### Plasmapherese

Bei der PPH werden Plasma und zelluläre Bestandteile des Bluts durch Filtration und Zentrifugation separiert, um danach das Plasma mittels einer albuminhaltigen Lösung oder Spenderplasma zu substituieren [20]. Im Rahmen einer PPH können Clearanceraten von maximal 50 ml/min erreicht werden, wodurch die Effizienz in der Toxinelimination als gering zu betrachten ist. Der Vorteil der Plasmapherese im Vergleich zu diskontinuierlichen Verfahren liegt in der Möglichkeit, auch Substanzen eliminieren zu können, die eine sehr hohe (> 95 %) Proteinbindung aufweisen und/oder sehr hohe Molekulargewichte (> 50 kDa) besitzen [3].

### Faktoren zur Optimierung der extrakorporalen Elimination

Prinzipiell wird die extrakorporale Clearance durch die folgenden Faktoren maximiert:

- hoher Blutfluss,
- hoher Dialysatfluss,
- hohe Ultrafiltrationsrate,
- große Membranoberfläche und
- lange Behandlungsdauer.

Die im Bereich der Dialyseliteratur beschriebenen Zusammenhänge sollten auch im toxikologischen Bereich gelten: Die Toxinclearance kann prinzipiell die niedrigste Flussrate nicht übersteigen; das bedeutet für die iHD den Blutfluss und für kontinuierliche Verfahren den Effluent-Fluss (Dialysatfluss ± Ultrafiltrat; [8]).

#### Merke

Höhere Blut- und Dialysatflüsse sowie eine größere Membranoberfläche und längere Behandlungsdauer erhöhen die extrakorporale Clearance.

## Initiierung und Management eines extrakorporalen Verfahrens bei Intoxikationen

Nachdem der Entschluss gefasst wurde, dass ein extrakorporales Verfahren von Vorteil zur Toxinelimination sein könnte, sollte die Prozedur sofern möglich, noch in der **Absorptionsphase**

**Abb. 2:**
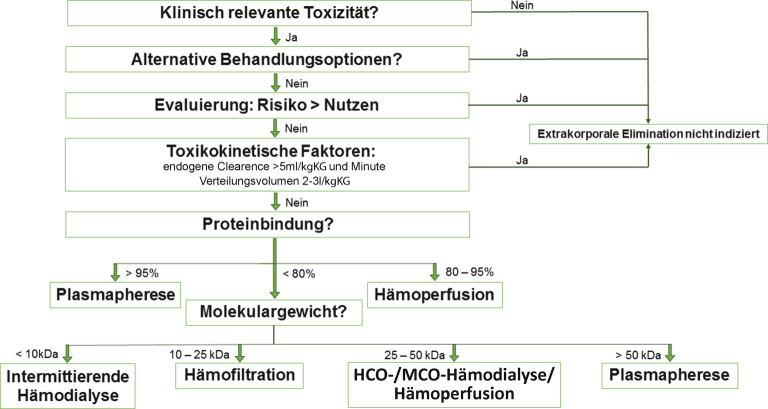
Flowchart zur Entscheidungshilfe im Rahmen der Evaluierung eines extrakorporalen Therapieverfahrens bei lebensbedrohlichen Intoxikation mit einer bekannten Substanz. *HCO* „high cut off“, *MCO* „medium cut off“. (Angelehnt an [3, 8])

Absorptionsphase

des Gifts, die bis zu 4 h dauern kann [21], beginnen, da sich in diesem Zeitraum noch der größte Anteil des Gifts im intravasalen Kompartiment befindet. Diesbezüglich eignet sich der **femorale Katheterzugangsweg**

femorale Katheterzugangsweg,

der keine Röntgenuntersuchung zur Überprüfung der korrekten Katheterlage erfordert. Zudem kommt das höhere Infektionsrisiko im Rahmen von Intoxikationen wahrscheinlich weniger zum Tragen, da eine Behandlung zumeist nicht länger als 3 Tage dauert [8]. Die Dauer der Therapie sollte an der klinischen Situation ausgerichtet werden und kann unter Umständen plasmaspiegelabhängig individuell angepasst werden. Hierbei ist zu erwähnen, dass intoxikierte PatientInnen ein niedriges Risiko für die Entwicklung eines Dysäquilibriumsyndroms aufweisen, da sie zumeist nicht an einer chronischen Nierenschädigung leiden. Die Abb. 2 zeigt eine übersichtliche Herangehensweise zur Entscheidungshilfe im Rahmen der Evaluierung eines extrakorporalen Therapieverfahrens bei lebensbedrohlichen Intoxikation.

### Merke

Die intermittierende Hämodialyse wird am häufigsten zur extrakorporalen Giftelemination eingesetzt.

## Schlussfolgerung

In den meisten Fällen sind supportive Maßnahmen ausreichend, um intoxikierte PatientInnen suffizient zu behandeln. In gewissen Situationen kann bei kritisch kranken, intoxikierten PatientInnen ein extrakorporales Verfahren, zumeist die iHD, toxinbedingte Komplikationen reduzieren oder gar verhindern. Die Empfehlungen der EXTRIP Workgroup sind zwar aufgrund der eingeschränkten Datenlage nicht endgültig, jedoch in den meisten Situationen sehr nützlich und die beste derzeit zu Verfügung stehende Entscheidungsgrundlage. Ein Verständnis der toxikokinetischen Eigenschaften und der grundlegenden Prinzipien der verfügbaren Therapiemodalitäten kann sehr hilfreich sein, um im intensivmedizinischen Setting und in der individuellen Therapieentscheidung den Benefit eines extrakorporalen Verfahrens abzuschätzen. Eine ergänzende telefonische Rücksprache mit einer Vergiftungsinformationszentrale ist allenfalls zu empfehlen.

## Fazit für die Praxis

- Eine effiziente Risikobeurteilung von intoxikierten PatientInnen ist essenziell, um den Nutzen eines extrakorporalen Therapieverfahrens abzuschätzen.
- Die Extracorporeal Treatments in Poisoning (EXTRIP) Workgroup veröffentlicht auf ihrer Homepage strukturierte und evidenzbasierte Handlungsempfehlungen zu den häufigsten Vergiftungen, die mittels extrakorporaler Verfahren behandelt werden können.
- Die extrakorporale Elimination eines Toxins wird hauptsächlich durch Molekulargewicht, Verteilungsvolumen und Proteinbindung beeinflusst.
- Die intermittierende Hämodialyse (iHD) mit Verwendung von modernen High-flux-Membranen ist das am häufigste eingesetzte extrakorporale Eliminationsverfahren im Rahmen von Intoxikationen.

## CME-Fragebogen

Welches der folgenden extrakorporalen Verfahren wird am häufigsten im Rahmen von Intoxikationen zur Elimination von Toxinen eingesetzt?

Plasmapherese

Extrakorporale Membranoxygenierung (ECMO)

Intermittierende Hämodialyse

Hämofiltration

Hämoperfusion

In welchen Fällen ist die Anwendung einer intermittierenden Hämodialyse bei Vergiftungen besonders effektiv?

Lipophile Substanzen wie Propofol

Großmolekulare Substanzen wie monoklonale Antikörper

Substanzen mit hoher Proteinbindung

Kleinmolekulare Substanzen mit hoher Wasserlöslichkeit

Substanzen mit großem Verteilungsvolumen

Welcher Vorteil wird der Plasmapherese im Vergleich zu anderen extrakorporalen Verfahren zugeschrieben?

Mithilfe der Plasmapherese können Substanzen mit niedriger Proteinbindung besser als mittels Hämodialyse entfernt werden.

Die Plasmapherese kann hilfreich sein, wenn das Toxin ein niedriges Molekulargewicht und eine niedrige Eiweißbindung besitzt.

Die Plasmapherese kann hilfreich sein, wenn das Toxin ein höheres Molekulargewicht und eine hohe Eiweißbindung besitzt.

Die Plasmapherese ist ineffektiv im Rahmen von Intoxikationen.

Bei der Plasmapherese können aufgrund des Wirkprinzips nur lipophile Substanzen eliminiert werden.

Welche Substanzen können durch Hämoperfusion mit einem Aktivkohlefilter *nicht* eliminiert werden?

Antibiotika

Tiergifte

Pflanzengifte

Alkohole

Lipophile Substanzen

Was gilt allgemein bei der Verwendung von extrakorporalen Verfahren im Rahmen von Intoxikationen?

Der Großteil der Intoxikationen sollte mit extrakorporalen Verfahren behandelt werden.

Vor der Verwendung eines extrakorporalen Verfahrens sollte ein strukturiertes Risikoassessment erfolgen.

Prinzipiell sollten komatöse, intoxikierte PatientInnen bevorzugt mit einem kontinuierlichen Verfahren behandelt werden.

Die intermittierende Hämodialyse bietet den Vorteil der hämodynamischen Stabilität.

Hämodynamische Instabilität ist eine Kontraindikation zur Verwendung eines extrakorporalen Verfahrens.

Warum spielt die endogene Clearance einer Substanz in der Abwägung eines extrakorporalen Verfahrens eine Rolle?

Eine niedrige endogene Clearance weist auf eine schlechte Verträglichkeit von extrakorporalen Verfahren hin.

Sobald ein extrakorporales Verfahren zur Anwendung kommt, ist die endogene Clearance irrelevant.

Eine höhere endogene Clearance bedeutet, dass extrakorporale Verfahren nicht notwendig sind.

Die endogene Clearance beeinflusst die Toxinkonzentration im Blut nicht.

Ein extrakorporales Verfahren ist indiziert, wenn die extrakorporale Clearance einen relevanten Teil zur Gesamtclearance beiträgt.

Von welchen Faktoren ist die extrakorporale Clearance am wenigsten abhängig?

Verteilungsvolumen

Plasmaproteinbindung

Molekulargewicht

Geschlecht

Wasserlöslichkeit

Was sollte beim Einsatz der intermittierenden Hämodialyse im Rahmen von Intoxikationen beachtet werden?

Die Temperatur der Dialysatflüssigkeit sollte unabhängig von der Art der Vergiftung immer schwanken.

Die Dauer der Hämodialysebehandlung sollte zumindest 12 h betragen.

Der Dialysezugang sollte aus Infektionsschutzgründen nicht in die V. femoralis gelegt werden.

PatientInnen mit Intoxikationen haben meist ein eher geringes Risiko für ein Dysäquilibriumsyndrom.

Bei Vergiftungen mit wasserlöslichen Substanzen ist die intermittierende Hämodialyse weniger effektiv als bei lipophilen Toxinen.

Wann ist der richtige Zeitpunkt, um mit einem extrakorporalen Therapieverfahren zu beginnen?

Erst wenn sich die gesamte Menge des Toxins im Blutkreislauf befindet, um effektiv zu dialysieren

Nach Möglichkeit noch in der Absorptionsphase des Gifts

Erst nach Fortbestehen der Intoxikation nach Anwendung anderer Eliminationsverfahren

Nach der Verabreichung eines effektiven Antidots

Bei intermittierender Hämodialyse sofortiger Beginn möglich, bei kontinuierlicher Dialyse erst nach hämodynamischer Stabilisierung

Welche Substanz ist *nicht* effektiv dialysierbar?

Lithium

Ethylenglykol

Metformin

Trizyklische Antidepressiva

Methanol
